# A Treatise on Practical Anatomy

**Published:** 1892-07

**Authors:** 


### Book Reviews.

A TREATISE ON PRACTICAL ANATOMY.—For students of anatomy. By Henry C. Boenning, M. D., Lecturer on Anatomy and Surgery in the Philadelphia School of Anatomy; Demonstrator of Anatomy in the Medico-Chirurgical College; Demonstrator of Anatomy in the Philadelphia Dental College; Lecturer on Disease in the Medico-Chirurgical College, etc. A work of 482 pages, and thoroughly illustrated with 198 wood engravings. Published by F. A. Davis, Philadelphia and London.

